# Paper 2: Performing rapid reviews

**DOI:** 10.1186/s13643-022-02011-5

**Published:** 2022-07-30

**Authors:** Valerie J. King, Adrienne Stevens, Barbara Nussbaumer-Streit, Chris Kamel, Chantelle Garritty

**Affiliations:** 1 The Center for Evidence-Based Policy, Oregon Health & Science University, Portland, Oregon, 97201 USA; 2 Epidemiology and Biostatistics, Unit Head, Public Health Agency of Canada, Ottawa, Canada; 3 Cochrane Austria, Danube University Krems, Krems, Austria; 4 Canadian Agency for Drugs and Technologies in Health, Ottawa, Canada; 5 Global Health & Guidelines Division, Public Health Agency of Canada, Ottawa, Canada

**Keywords:** Rapid review, Systematic review, Technology assessment, Evidence-based medicine

## Abstract

**Background:**

Health policy-makers must often make decisions in compressed time frames and with limited resources. Hence, rapid reviews have become a pragmatic alternative to comprehensive systematic reviews. However, it is important that rapid review methods remain rigorous to support good policy development and decisions. There is currently little evidence about which streamlined steps in a rapid review are less likely to introduce unacceptable levels of uncertainty while still producing a product that remains useful to policy-makers.

**Methods:**

This paper summarizes current research describing commonly used methods and practices that are used to conduct rapid reviews and presents key considerations and options to guide methodological choices for a rapid review.

**Results:**

The most important step for a rapid review is for an experienced research team to have early and ongoing engagement with the people who have requested the review. A clear research protocol, derived from a needs assessment conducted with the requester, serves to focus the review, defines the scope of the rapid review, and guides all subsequent steps. Common recommendations for rapid review methods include tailoring the literature search in terms of databases, dates, and languages. Researchers can consider using a staged search to locate high-quality systematic reviews and then subsequently published primary studies. The approaches used for study screening and selection, data extraction, and risk-of-bias assessment should be tailored to the topic, researcher experience, and available resources. Many rapid reviews use a single reviewer for study selection, risk-of-bias assessment, or data abstraction, sometimes with partial or full verification by a second reviewer. Rapid reviews usually use a descriptive synthesis method rather than quantitative meta-analysis. Use of brief report templates and standardized production methods helps to speed final report publication.

**Conclusions:**

Researchers conducting rapid reviews need to make transparent methodological choices, informed by stakeholder input, to ensure that rapid reviews meet their intended purpose. Transparency is critical because it is unclear how or how much streamlined methods can bias the conclusions of reviews. There are not yet internationally accepted standards for conducting or reporting rapid reviews. Thus, this article proposes interim guidance for researchers who are increasingly employing these methods.

**Supplementary Information:**

The online version contains supplementary material available at 10.1186/s13643-022-02011-5.

## Background

### Introduction

Health policy-makers and other stakeholders need evidence to inform their decisions. However, their decisions must often be made in short time frames, and they may have other resource constraints, such as the available budget or personnel [1–6]. Rapid reviews are increasingly being used and are increasingly influential in the health policy and system arena [3, 7–10]. One needs assessment [11] showed that policy-makers want evidence reviews to answer the right question, be completed in days to weeks, rather than months or years, be accurate and reproducible, and be affordable.

As much as policy-makers may desire faster and more efficient evidence syntheses, it is not yet clear whether rapid reviews are sufficiently rigorous and valid, compared to systematic reviews which are considered the “gold standard” evidence synthesis, to inform policy [12]. Only a few empirical studies have compared the findings of rapid reviews and systematic reviews on the same topic, and their results are conflicting and inconclusive, leaving questions about the level of bias that may be introduced because of rapid review methods [7, 13–19].

A standardized or commonly agreed-upon set of methods for conducting rapid reviews had not existed until recently, [1, 9, 14, 20–23] and while there is little empiric evidence on some of the standard elements of systematic reviews, [24] those standards are well articulated [25, 26]. A minimum interim set of standards has was developed by the Cochrane Rapid Reviews Methods Group [1, 2] to help guide rapid review production during the SARS-CoV-19 pandemic, and other researchers have proposed methods and approaches to guide rapid reviews [5, 21, 22, 27–36].

This article gives an overview of potential ways to produce a rapid review while maintaining a synthesis process that is sufficiently rigorous, yet tailored as needed, to support health policy-making. We present options for common methods choices, summarized from descriptions and evaluations of rapid review products and programs in Table 1, along with key considerations for each methodological step.

**Table 1:** Common methods, approaches, and key considerations for the steps in a rapid review

Review step	Commonly employed methods and approaches	Key considerations
Needs assessment, topic selection, and topic refinement	•Most use standard intake processes, involving the requester, to refine the topic, obtain clarity on purpose(s), and determine whether rapid review is a suitable method •Total production timeline generally 1 to 4 months	•Work with requester to ascertain intended purpose, scope, and timeline and ensure that proposed approach fits intended purpose. Streamline the request to focus on a limited number of high priority questions and outcomes •A preliminary literature search can help to inform conversations with requester and to scope the review •Map the mandate to timeline and deliverables
Protocol development	•A protocol is commonly prepared, serving as a point of reference to avoid (or document) deviations, but is usually not formally registered •Producers typically use a PICO format and develop key questions iteratively with requesters	•Consider registering the protocol with PROSPERO (https://www.crd.york.ac.uk/PROSPERO/) or the Open Science Framework (https://osf.io/) and include “rapid review” or a similar term in the title •Use PRISMA-P [37] (http://www.prisma-statement.org/Extensions/Protocols) reporting items to guide protocol development and the PRISMA [38] statement for systematic reviews (http://www.prisma-statement.org/PRISMAStatement/) for review reporting and to track the overall process and information flow
Literature search	•Many rapid reviews are based on searches of the PubMed/MEDLINE, Cochrane Library, and Embase databases •Most entail a search of two or more databases, with common limits being date, language (generally English only), and study design; geographical limits may be used to enhance applicability •Some level of gray literature searching is common, but contact with authors is uncommon	•Tailor the selection of literature databases to the topic. Addition of a gray literature search depends on the topic, purpose, and timeline •Use a staged search to first identify existing systematic reviews and then studies with other designs that will provide the most rigorous evidence to answer the question •Peer review of the search strategy, using a tool such as the PRESS checklist, can help to optimize the search strategy [39]
Screening and study selection	•Approaches are highly variable, with about half of rapid reviews using a single reviewer, with or without verification by a second reviewer	•Choose the approach for study screening and selection according to requirements of the review and resources available •In lieu of dual screening and selection, reasonable approaches involve using a single experienced reviewer for application of inclusion criteria and two reviewers for application of exclusion criteria or using one person for screening with verification of a subset of records by another
Data extraction	•Approaches vary, but data extraction by a single reviewer, with or without verification, is the most common method	•Similar to the situation for screening, the number of independent reviewers varies, but a reasonable approach is to use a single reviewer to extract data, with a second reviewer checking at least a 10% random sample of extractions for accuracy. Use of dual performance or checking may be needed more for extraction of quantitative results than for extraction of descriptive study information •Limit extraction to key study characteristics and outcomes
Risk-of-bias assessment	•For most rapid reviews, some risk-of-bias or quality assessment of included studies is conducted by a single reviewer, with or without verification	•The choice of appraisal instrument varies, with both standard and customized approaches in use •An approach similar to that for data extraction can be used (i.e., single reviewer, with verification by a second reviewer)
Synthesis	•Narrative summaries are common, with meta-analysis performed only infrequently •Final reports often include implications, recommendations for policy, and discussion of research limitations	•An iterative approach to the synthesis process can involve post hoc protocol adjustments •The quality of the body of evidence and the strength of any recommendations can be assessed using an approach such as the GRADE system (http://www.gradeworkinggroup.org/) •The limitations of the review should be discussed and cautious conclusions provided
Report production and dissemination	•Peer review is common but is often performed internally •Reports are often disseminated beyond the original requester but are infrequently published in the peer-reviewed literature	•Software tools can help to automate and track review steps. Systematic Review Toolbox is a searchable database of software tools to support evidence synthesis tasks (http://systematicreviewtools.com/) •Standardization of processes and templates aids in production of the report and enhances transparency of the review

*GRADE*, Grading of Recommendations Assessment, Development and Evaluation; *PICO*, population, intervention, comparator, and outcome; *PRESS*, peer review of electronic search strategies; *PRISMA-P*, Preferred Reporting for Systematic Reviews and Meta-Analyses Protocols; *PRISMA*, Preferred Reporting Items for Systematic Reviews and Meta-Analyses

## Methods

The World Health Organization (WHO) published *Rapid reviews to strengthen health policy and systems: a practical guide* [5] in 2017. The initial work for this article was completed as a chapter for that publication and included multiple literature searches and layers of peer review to identify important studies and concepts. We conducted new searches using Ovid MEDLINE, the Cochrane Library’s methodology collection, and the bibliography of studies maintained by the Cochrane Rapid Reviews Methods Group, to identify articles, including both examples of rapid reviews and those on rapid review methodology, published after the publication of the WHO guide. We have not attempted to perform a comprehensive identification or catalog of all potential articles on rapid reviews or examples of reviews conducted with these methods. As this work was not a systematic review of rapid review methods, we do not include a flow of articles from search to inclusion and have not undertaken any formal critical appraisal of the articles we did include.

## Results

### Needs assessment, topic selection, and topic refinement

Rapid reviews are typically conducted at the request of a particular decision-maker, who has a key role in posing the question, setting the parameters of the review, and defining the timeline [40–42]. The most common strategy for completing a rapid review within a limited time frame is to narrow its scope. This can be accomplished by limiting the number of questions, interventions, and outcomes considered in the review [13, 15]. Early and continuing engagement of the requester and any other relevant stakeholders is critical to understand their needs, the intended use of the review, and the expected timeline and deliverables [15, 28, 29, 40–42]. Policy-makers and other requesters may have vaguely defined questions or unrealistic expectations about what any type of review can accomplish [41, 42]. A probing conversation or formal needs assessment is the critical first step in any knowledge synthesis approach to determine the scope of the request, the intended purpose for the completed review, and to obtain a commitment for collaboration over the duration of the project [28, 30, 41]. Once the request and its context are understood, researchers should fully develop the question(s), including any needed refinement with the requester or other stakeholders, before starting the project [5]. This process can be iterative and may require multiple contacts between the reviewers and the requester to ensure that the final rapid review is fit for its intended purpose [41, 42]. In situations where a definitive systematic review might be needed, it may be useful to discuss with the requester the possibility of conducting a full systematic review, either in parallel or serially with the rapid review [43].

### Protocol development

A research protocol clearly lays out the scope of the review, including the research questions and the approaches that will be used to conduct the review [44]. We suggest using the Preferred Reporting Items for Systematic Review and Meta-Analysis Protocols (PRISMA-P) 2015 statement for guidance [37]. Most reviewers use the PICO format (population, intervention, comparator, outcome), with some adding elements for time frame, setting, and study design. The PICO elements help to define the research questions, and the initial development of questions can point to needed changes in the PICO elements. For some types of research questions or data, other framework variations such as SPICE (setting, perspective, intervention, comparison, evaluation) may be used, although the PICO framework can generally be adapted [45]. Health services and policy research questions may call for more complex frameworks [5]. This initial approach assists both researchers and knowledge users to know what is planned and enables documentation of any protocol deviations; however, the customized and iterative nature of rapid reviews means that some flexibility may be required. Some rapid review producers include the concept of methods adjustment in the protocol itself [46, 47]. However, changes made beyond the protocol stage and the rationale for making them must be transparent and documented in the final report.

The international prospective register of systematic reviews (PROSPERO) [44] (https://www.crd.york.ac.uk/PROSPERO/) accepts registration of protocols that include at least one clinically or patient-relevant outcome. The Open Science Framework (OSF) [48] platform (https://osf.io/) also accepts protocol registrations for rapid reviews. We advise protocol submitters to include the term “rapid review” or another similar term in the registered title, as this will assist tracking the use, validity, and value of rapid reviews [1]. Protocol registration helps to decrease research waste and allows both requesters and review authors to avoid duplication. Currently, most rapid review producers report using a protocol, but few register their protocols [13, 17].

### Literature search

Multiple authors have conducted inventories of the characteristics of and methods used for rapid reviews, including the broad categories of literature search, study selection, data extraction, and synthesis steps [13, 15, 17, 20, 24, 49]. PRISMA (Preferred Reporting Items for Systematic Reviews and Meta-Analyses) standards call for documentation of the full search strategy for all electronic databases used [38]. Most published rapid reviews search two or more databases, with PubMed, Embase, and the Cochrane Library mentioned frequently [13, 17, 20, 49]. Rapid reviews often streamline systematic review methods by limiting the number of databases searched and the search itself by date, language, geographical area, or study design, and some rapid reviews search only for existing systematic reviews [13, 15, 17, 20, 49, 50]. Other rapid reviews use a layered searching approach, identifying existing systematic reviews and then updating them with a summary of more recent eligible primary studies [13, 15, 18, 20, 36]. Studies of simplified search strategies have generally demonstrated acceptable retrieval characteristics for most types of rapid review reports [51, 52]. Searching the reference lists of eligible studies (sometimes known as the “snowballing” technique) and searching the gray literature (i.e., reports that are difficult to locate or unpublished) are done in about half of published rapid reviews and may be essential for certain topics [13, 15, 20, 49]. However, rapid reviews seldom report contact with authors and other experts to identify additional unpublished studies [13, 15, 20, 49]. One study found that peer review of the search strategy, using a tool such as the PRESS (peer review of electronic search strategies) checklist, [39] was reported in 38% of rapid reviews, but that it was usually performed internally rather than by external information specialist reviewers [13]. Peer review of search strategies has been reported to increase retrieval of relevant records, particularly for nonrandomized studies [53].

### Screening and study selection

Methodological standards for systematic reviews generally require independent screening of citations and abstracts by at least two researchers to arrive at a set of potentially eligible references, which are in turn subjected to dual review in full-text format to arrive at a final inclusion set. Rapid reviews often streamline this process, with up to 40% using a single researcher at each stage [13, 15, 17, 18, 20, 49]. Some rapid reviews report verification of a sample of the articles by a second researcher or, occasionally, use of full dual screening by two independent researchers [13, 17, 20, 49]. One methodological study reported that single screener selection missed an average of 5% of eligible studies, ranging from 3% for experienced reviewers and 6% for those with less experience [54]. If time and resources allow, we recommend that dual screening of all excluded studies, at both the title and full-text stages, be used to minimize the risk of selection bias through the inappropriate exclusion of relevant studies. However, there is some evidence that the use of a single experienced reviewer for particular topics may be sufficient [18, 46, 54].

### Data extraction

As with citation screening and study selection, the number of independent reviewers who extract study data for a rapid review can vary. One study found that the most common approach is single-reviewer extraction (41%), although another 25% report verification of a sample by a second reviewer and nearly as many used dual extraction [13]. A more recent study reported that only about 10% of rapid reviews examined reported dual data extraction, although nearly twice as many simply did not report this feature [17]. Data abstraction generally includes PICO elements, although data abstraction was often limited by the scope of the review, and authors were contacted for missing data very infrequently [13].

### Risk-of-bias assessment

Risk-of-bias assessment, sometimes called critical appraisal or methodological quality appraisal, examines the quality of the methods employed for each included study and is a standard element of systematic reviews [25]. The vast majority of rapid review producers perform some type of critical appraisal [17, 20]. Some rapid reviews report the use of a single assessor with verification of a sample of study assessments by another assessor [17, 49]. There is no consensus as to which risk-of-bias assessment tools should be used, although most reviews use study design-specific instruments (e.g., an instrument designed for randomized controlled trials (RCTs) if assessing RCTs) intended for assessing internal validity [13, 20].

### Knowledge synthesis

Nearly all rapid review producers conduct a descriptive synthesis (also often called a narrative synthesis) of results, but a few perform additional meta-analyses or economic analyses [13, 17, 20]. The synthesis that is conducted is often limited to a basic descriptive summary of studies and their results, rather than the full synthesis that is recommended for systematic reviews [26]. Most rapid reviews present conclusions, recommendations, or implications for policy or clinical practice as another component of the synthesis. Multiple experts also recommend that rapid reviews clearly describe and discuss the potential limitations arising from methodological choices [5, 9, 13, 15, 23].

Many systematic review producers use the Grading of Recommendations Assessment, Development and Evaluation (GRADE) system [55] (http://www.gradeworkinggroup.org/) to rate the certainty of the evidence about health outcomes. Guideline developers and others who make recommendations or policy decisions use GRADE to rate the strength of recommendations based on that evidence. The GRADE evidence to decisions (EtD) framework has also been used to help decision-makers developing health system and public health [56] and coverage [57] policies. Rapid review authors can also employ GRADE to rate the certainty of synthesized evidence and develop policy implications for decision-makers if time and resources permit. However, the GRADE system works best for interventions that have been subject to RCTs and where there is at least one meta-analysis to provide a single estimate of effect.

### Report production and dissemination

Standard templates for each stage of the review, from protocol development to report production, can assist the review team in performing each step efficiently. Use of a report template, with minimum methodological standards, reporting requirements, and standard report sections, can assist the producer in streamlining production of the report and can also enhance transparency [15, 20, 28, 40]. An extension of the PRISMA statement for rapid reviews is under development and has been registered with the EQUATOR Network [58]. Until it is available, the PRISMA checklist for systematic reviews can serve as a reporting template to increase the transparency of rapid reviews [8, 40, 59].

Research about review formatting and presentation of rapid review is being conducted, but it is likely that the forms employed and tested will need to be adapted to the individual requester and stakeholder audiences [47]. Khangura and colleagues [28] have presented a figure showing formatted sections of a sample report, and many other rapid review producers have examples of reports online that can serve as formatting examples. In addition, findings from evidence summary presentation research for decision-makers in low- and middle-income countries can be translated into other settings [60, 61].

Most rapid review producers conduct some form of peer review for the resulting reports, but such review is often internal and may include feedback from the requester [13]. Most producers disseminate their reports beyond the requester, but dissemination varies by the sensitivity or proprietary nature of the product [13, 20]. When reports are disseminated, it is common for them to be posted online, for example, at an organizational website [13, 20].

### Operational considerations

Evaluations and descriptions of research programs that produce rapid reviews typically include some helpful pragmatic and operational considerations for undertaking a rapid review or developing a rapid review program [5, 15, 18, 27–29, 31, 36, 40, 62, 63]. Highly experienced, permanent staff with the right skill mix, including systematic reviewers, information specialists, methodologists, and content experts [15, 18, 30, 40, 49], are essential. It is time-consuming to assemble staff on a per-project basis, so the presence of an existing team (which may only do rapid reviews or may also do systematic reviews or other research) with review infrastructure already in place allows projects to get off to a quick start. The existence of a dedicated team also creates the potential to build relationships with requesters and to cultivate mutual trust. Staff with experience conducting systematic reviews will be familiar with standard methods and may be alert to any needed protocol changes as the review proceeds [49]. The rapid review team must understand the methodological implications of decisions taken and must convey these implications to the requesters, to allow them to understand the caveats and potential limitations. Continuing relationships and longer-term contracting with requesters, to allow for a quick start and “good faith” initiation of work before a contract is in place, can speed the early development stages [31, 40]. It is important for rapid review producers to confirm that the choices they make to streamline the review are acceptable to the requester [41]. Whether it is a decision to limit the scope to a single intervention or outcome, restrict the literature search to existing systematic reviews, or forgo a meta-analysis, the knowledge user must be aware of the implications of streamlining decisions [15, 27, 31, 41]. Some programs also emphasize the need for follow-up with review requesters to develop the relationship and continuously improve knowledge products [28, 63]. Although it is beyond the scope of this article, we note that both systematic and rapid review producers are currently using various automated technologies to speed review production. There are examples of tools to help search for references, screen citations, abstract data, organize reviews, and enhance collaboration, but few evaluations of their validity and value in report production [64, 65]. The Systematic Review Toolbox [66] (http://systematicreviewtools.com/) is an online searchable database of tools that can help perform tasks in the evidence synthesis process.

Table 1 summarizes the commonly described approaches and key considerations for the major steps in a rapid review that are discussed in detail in the preceding sections.

### Suggested approaches to rapid reviews

The previous sections have summarized the numerous approaches to conducting rapid reviews. Abrami and colleagues [27] summarized several methods of conducting rapid reviews and developed a brief review checklist of considerations and recommendations, which may serve as a useful parallel to Table 2. A “one-size-fits-all” approach may not be suitable to cover the variety of topics and requester needs put forward. Watt and colleagues [9] observed over a decade ago, “It may not be possible to validate methodological strategies for conducting rapid reviews and apply them to every subject. Rather, each topic must be evaluated by thorough scoping, and appropriate methodology defined.” Plüddemann and colleagues [23] advocated for a flexible framework for what they term “restricted reviews,” with a set of minimum requirements and additional steps to reduce the risk of bias when time and resources allow. Thomas, Newman, and Oliver [29] noted that it might be more difficult to apply rapid approaches to questions of social policy than to technology assessment, in part because of the complexity of the topics, underlying studies, and uses of these reviews. The application of mixed methods, such as key informant interviews, stakeholder surveys, primary data, and policy analysis, may be required for questions with a paucity of published literature and those involving complex subjects [29]. However, rapid review producers should remain aware that streamlined methods may not be appropriate for all questions, settings, or stakeholder needs, and they should be honest with requesters about what can and cannot be accomplished within the timelines and resources available [31]. For example, a rapid review would likely be inappropriate as the foundation for a national guideline on cancer treatment due to be launched 5 years in the future. A decision tool, STARR (SelecTing Approaches for Rapid Reviews) has been published by Pandor and colleagues [67] to help guide decisions about interacting with report requesters, making informed choices regarding to the evidence base, methods for data extraction and synthesis, and reporting on the approaches used for the report.

**Table 2:** Interim guidance for rapid reviews

1.Engage with the review requester early and throughout the review process to understand needs and expectations and collaborate with the requester in making decisions about how to approach the review
2.Use a team experienced in doing systematic reviews to conduct the rapid review
3.Develop a protocol, including PICO (population, intervention, comparator, outcome) elements, key questions, and the planned approach, to guide the review and to track any changes that are made as the review progresses (and their rationale). Protocols registration is strongly encouraged
4.Search at least two electronic databases for most topics; use a targeted gray literature search if the topic is not well addressed in published articles
5.If timeline and resources allow, use two reviewers for study selection
6.Perform data extraction and risk-of-bias assessment using one researcher; if time and resources allow, a sample of articles should be checked by a second one
7.Consider the use of innovative technologies that can help to make particular review steps more efficient
8.In conducting the knowledge synthesis, include both a typical results component (with description of included studies, their results, reasons for any differences in results across studies, and the quality of the evidence from those studies, perhaps with GRADE (Grading of Recommendations Assessment, Development and Evaluation) rating for the overall quality of evidence) and a discussion component describing limitations of the evidence and the review, overall conclusions, recommendations, and implications for policy- and decision-makers
9.When possible, obtain peer review and use feedback from the requester and other stakeholders to inform and improve future knowledge synthesis
10.Consult with the requester about the best report format and presentation that will support use of the review and subsequent decision-making. Use report templates and make the report publically available whenever possible

Tricco and colleagues [21] conducted an international survey of rapid review producers, using a modified Delphi ranking to solicit opinions about the feasibility, timeliness, comprehensiveness, and risk of bias of six different rapid review approaches. Ranked best in terms of both risk of bias and feasibility was “approach 1,” which included published literature only, based on a search of one or more electronic databases, limited in terms of both date and language. With this approach, a single reviewer conducts study screening, and both data extraction and risk-of-bias assessment are done by a single reviewer, with verification by a second researcher. Other approaches were ranked best in terms of timeliness and comprehensiveness, [21] representing trade-offs that review producers and knowledge users may want to consider. Because the survey report was based on expert opinion, it did not provide empirical evidence about the implications of each streamlined approach [21]. However, in the absence of empirical evidence, it may serve as a resource for rapid review producers looking to optimize one of these review characteristics. Given that evidence regarding the implications of methodological decisions for rapid reviews is limited, we have developed interim guidance for those conducting rapid reviews (Table 2).

## Discussion

Rapid reviews are being used with increasing frequency to support clinical and policy decisions [6, 22, 34]. While policymakers are generally willing to trade some certainty for speed and efficiency, they do expect rapid reviews to come close to the validity of systematic reviews [51]. There is no universally accepted definition of a rapid review [2]. This lack of consensus is, in part, related to the grouping of products with different purposes, audiences, timelines, and resources. Although we have attempted to summarize the major choices available to reviewers and requesters of information, there are few empiric data to guide these choices. We may have missed examples of rapid reviews and methodological research that could add to the conclusions of this paper. However, our approach to this work has been pragmatic, much like a rapid review itself, and is based on our international experience as researchers involved in the Cochrane Rapid Reviews Methods Group, as well as authors who participated in the writing and dissemination of *Rapid reviews to strengthen health policy and systems: a practical guide* [5]. This paper has, in addition, been informed by our research about rapid reviews and our collective work across several groups that conduct rapid reviews [1, 68]. The Cochrane Rapid Review Methods Group also conducted a methods opinion survey in 2019 and released interim recommendations to guide Cochrane rapid reviews during the SARS-CoV-2 pandemic [2]. These recommendations are specific to the needs of Cochrane reviews and offer more detailed guidance for rapid review producers than those presented in this paper. We encourage readers to sign up for the Cochrane Rapid Reviews Methods Group newsletter on the website (https://methods.cochrane.org/rapidreviews/) and to check the list of methodological publications which is updated regularly to continue to learn about research pertinent to rapid reviews [68].

## Conclusions

We have summarized the rapid review methods that can be used to balance timeliness and resource constraints with a rigorous knowledge synthesis process to inform health policy-making. Interim guidance suggestions for the conduct of rapid reviews are outlined in Table 2. The most fundamental key to success is early and continuing engagement with the research requester to focus the rapid review and ensure that it is appropriate to the needs of stakeholders. Although the protocol serves as the starting point for the review, methodological decisions are often iterative, involving the requester. Any changes to the protocol should be reflected in the final report. Methods can be streamlined at all stages of the review process, from search to synthesis, by limiting the search in terms of dates and language; limiting the number of electronic databases searched; using one reviewer to perform study selection, risk-of-bias assessment, and data abstraction (often with verification by another reviewer); and using a descriptive synthesis rather than a quantitative summary. Researchers need to make transparent methodological choices, informed by stakeholder input, to ensure that the evidence review is fit for its intended purpose. Given that it is not clear how these choices can bias a review, transparency is essential. We are aware that an increasing number of journals publish rapid reviews and related evidence synthesis products, which we hope will further increase the availability, transparency, and empiric research base for progress on rapid review methodologies.

## Supplementary Information

**Additional file 1.** Funding.

## Abbreviations

EQUATOR: Enhancing the QUAlity and Transparency Of health Research
GRADE: Grading of Recommendations Assessment, Development and Evaluation
PICO: Population, intervention, comparator, outcomes
PRISMA: Preferred Reporting Items for Systematic Reviews and Meta-Analyses
PRISMA-P: Preferred Reporting Items for Systematic Reviews and Meta-Analyses-Protocols
RCT: Randomized controlled trial
SPICE: Setting, Perspective, Intervention, Comparator, Evaluation
STARR: SelecTing Approaches for Rapid Reviews
PRESS: Peer review of electronic search strategies
WHO: World Health Organization
